# Transforming healthcare through a digital revolution: A review of digital healthcare technologies and solutions

**DOI:** 10.3389/fdgth.2022.919985

**Published:** 2022-08-04

**Authors:** Nithesh Naik, B. M. Zeeshan Hameed, Nilakshman Sooriyaperakasam, Shankeeth Vinayahalingam, Vathsala Patil, Komal Smriti, Janhavi Saxena, Milap Shah, Sufyan Ibrahim, Anshuman Singh, Hadis Karimi, Karthickeyan Naganathan, Dasharathraj K. Shetty, Bhavan Prasad Rai, Piotr Chlosta, Bhaskar K. Somani

**Affiliations:** ^1^Department of Mechanical and Industrial Engineering, Manipal Institute of Technology, Manipal Academy of Higher Education, Manipal, Karnataka, India; ^2^iTRUE (International Training and Research in Uro-Oncology and Endourology) Group, Manipal, Karnataka, India; ^3^Department of Urology, Father Muller Medical College, Mangalore, Karnataka, India; ^4^Department of Mechanical Engineering, University of Moratuwa, Moratuwa, Sri Lanka; ^5^Department of Oral and Maxillofacial Surgery, Radboud University, Nijmegen, Netherlands; ^6^Department of Oral Medicine and Radiology, Manipal College of Dental Sciences, Manipal, Manipal Academy of Higher Education, Manipal, Karnataka, India; ^7^Robotics and Urooncology, Max Hospital and Max Institute of Cancer Care, New Delhi, India; ^8^Kasturba Medical College, Manipal Academy of Higher Education, Manipal, Karnataka, India; ^9^Department of Urology, Kasturba Medical College, Manipal, Manipal Academy of Higher Education, Manipal, Karnataka, India; ^10^Manipal College of Pharmaceutical Sciences, Manipal Academy of Higher Education, Manipal, Karnataka, India; ^11^Frontline Hospital and Research Centre, Trichy, Tamil Nadu, India; ^12^Department of Data Science and Computer Applications, Manipal Institute of Technology, Manipal Academy of Higher Education, Manipal, Karnataka, India; ^13^Department of Urology, Freeman Hospital, Newcastle upon Tyne, United Kingdom; ^14^Department of Urology, Jagiellonian University in Krakow, Kraków, Poland; ^15^Department of Urology, University Hospital Southampton NHS Trust, Southampton, United Kingdom

**Keywords:** healthcare, telemedicine, digital healthcare, artificial intelligence, blockchain

## Abstract

The COVID-19 pandemic has put a strain on the entire global healthcare infrastructure. The pandemic has necessitated the re-invention, re-organization, and transformation of the healthcare system. The resurgence of new COVID-19 virus variants in several countries and the infection of a larger group of communities necessitate a rapid strategic shift. Governments, non-profit, and other healthcare organizations have all proposed various digital solutions. It's not clear whether these digital solutions are adaptable, functional, effective, or reliable. With the disease becoming more and more prevalent, many countries are looking for assistance and implementation of digital technologies to combat COVID-19. Digital health technologies for COVID-19 pandemic management, surveillance, contact tracing, diagnosis, treatment, and prevention will be discussed in this paper to ensure that healthcare is delivered effectively. Artificial Intelligence (AI), big data, telemedicine, robotic solutions, Internet of Things (IoT), digital platforms for communication (DC), computer vision, computer audition (CA), digital data management solutions (blockchain), digital imaging are premiering to assist healthcare workers (HCW's) with solutions that include case base surveillance, information dissemination, disinfection, and remote consultations, along with many other such interventions.

## Introduction

The recent outbreak of the COVID-19 pandemic has had a significant impact on socio-economic, sustainability, and healthcare systems across the world. The disease is highly contagious and causes a range of symptoms (1) including dry cough, diarrhea, headache, fever, sore throat, and conjunctivitis, amongst others. At the end of May 2021, digital data repositories have claimed around 3,565,021 mortalities, and more than 171 million morbidities globally (2). The number of critically ill patients in developed countries is exceeding the critical care capacity (3, 4) and low-income and middle-income countries (LMICs) are even more at risk due to the lack of medical infrastructure (5) to manage the surge of the disease. The availability of intensive critical care (ICU) for severely ill patients is also a major deciding factor in mortality rates in underdeveloped and developing countries. Preventive measures and precautions should be taken in developing countries before it exceeds their healthcare capacity. The need for suitable preventive measures to mitigate the impact of the pandemic is in the spotlight. The conventional preventive methods including social distancing, quarantine, lockdowns, and safety gear are all under practice. However, the lockdowns and quarantines have led to psychological distress in individuals (6–9) and economic recession (6, 7, 10, 11).

Information Technology (IT) sector is primarily gaining attention because digital health technologies can provide effective measures to tackle the ongoing challenge (12–14). Artificial intelligence (AI), cloud data solutions, real-time tracking and monitoring systems, telemedicine powered by 5G, and robotic technologies can provide innovative solutions for front-line protection, accelerated detection, infectious risk management to reduce morbidity and mortality rates, and their consequences. World Health Organization(WHO) has developed a practical guide that is to be used by national authorities to develop and update their COVID-19 responses (15). Following these guidelines, various national health ministries have advocated the promotion and prevention of disease spread while embracing a cascade of healthcare measures (16). This review discusses the related healthcare models presently practiced in developing countries, and advances that can be potentially deployed in LMICs. The substantial exposure to the current advancements in digital health technologies could incentivize new beginnings in technological ventures and encourage the adoption of the latest healthcare models. The review synthesizes global trends in digital healthcare with a siloed system approach to replicate the process within individual countries.

## The necessity of digital healthcare

**Figure 1:**
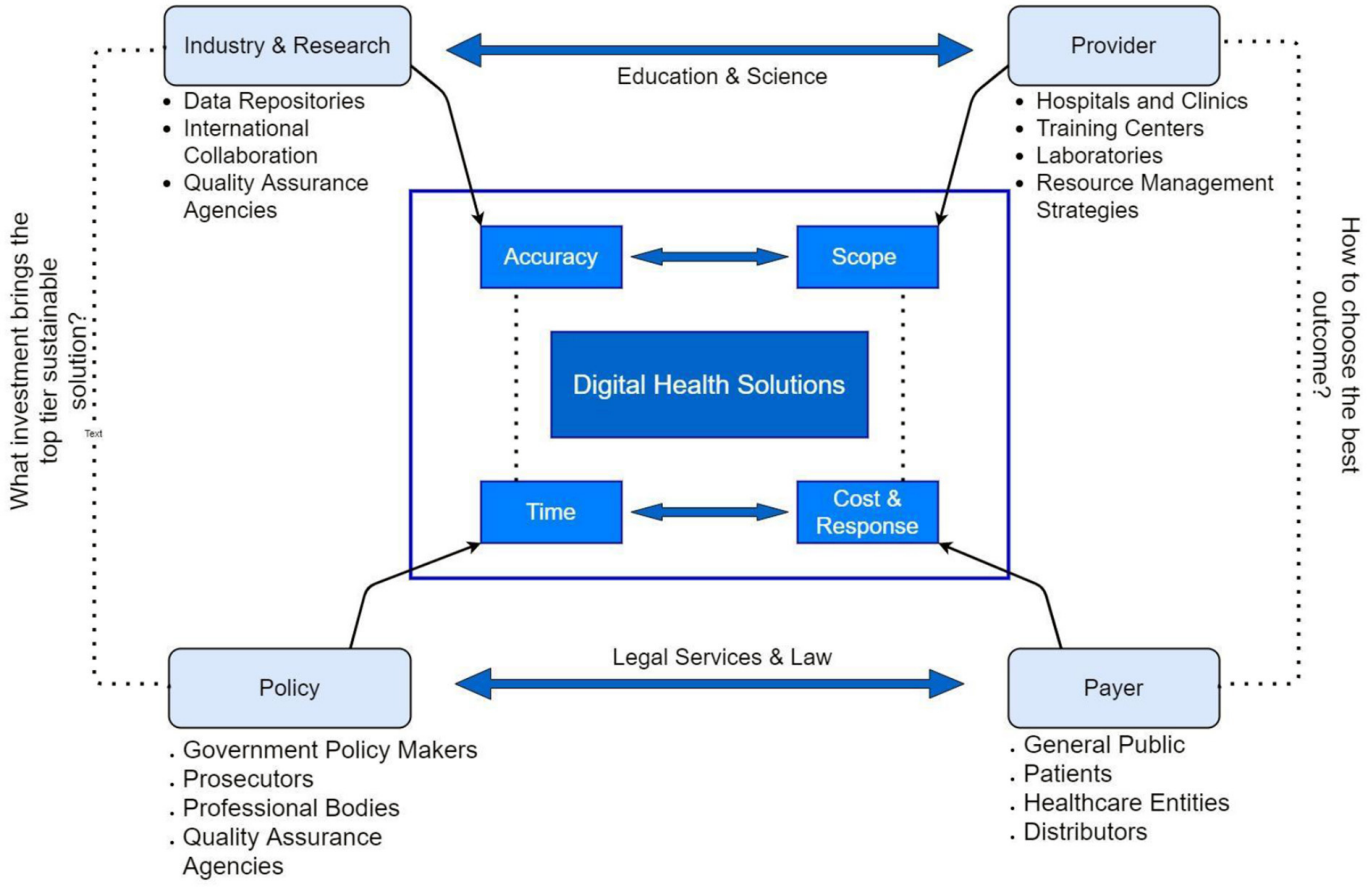
Governing factors on personalized health responses.

The healthcare systems in LMICs are suffering from a lack of cutting-edge technologies that are available in developed countries. The private healthcare service providers often play a competitive role at par with the government sector in providing state-of-the-art medical facilities (17). The long wait for these consultations and advanced procedures in government healthcare facilities creates a space for private sector healthcare setups to provide quicker and often better services with higher payments. The low-income group, especially in villages are suffering to afford timely medical care and quality healthcare services. However, several factors that govern the overall performance of the health sector in a country are vividly discussed in Figure 1. While the current pandemic is de-normalizing healthcare management globally, LMICs are facing a severe threat. Improvement in world-class training, deployment of skillful practitioners, external inbound investment, broadening mindset of inland naive users, demands of high moral values in the country, ability to possess digital devices, willingness to adopt changes, improved educational facilities and strengthened inter-country fraternity against invisible enemy creates a thrust for LMICs to adopt and adapt the digital health care models.

## Digital healthcare models

### Tracking suspected subset of individuals *via* open-source web solutions

Studies showed that the number of new infectious cases are doomed by a single infectious person (18, 19) and the infected person could either be symptomatic or asymptomatic (20, 21). Hence, identifying the infected patient, tracing their contact, to contain the transmission, is crucial. A centralized tracking technology, i.e., the Pan-European Privacy-Preserving Proximity Tracing (PEPP-PT) (22), is facilitating the creation of a social graph of individuals and their interactions. This centralized approach traces geological locations, whereabouts, and close contacts, and stores them in central repositories for the concerned authorities to access and monitor the sensitive information. However, civil rights authorities stand against this centralized approach (23), as law enforcement entities and government departments can access the information for future surveillance. Therefore, they are emphasizing de-centralized data tracing, i.e., Decentralized privacy-preserving proximity tracing (DP-3T) (22). On the other hand, many European countries are interested in short range tracking technologies such as Bluetooth technology (Bluetooth tracking) to fit this model. Generally, contact tracers with sensitive details including CCTV footage, location tracking, credit card transactions are more likely incompatible with privacy-minded protocols. Thus, Bluetooth tracking is apparently made alluring. Bluetooth's “*Handshaking*” is enough to identify close contacts and contact duration. Meanwhile, Google and Apple have announced that they will provide technological support for de-centralized contact tracing solutions (24).

### District health information software (DHIS2) digital toolkit

District Health Information Software 2 (DHIS2) has released digital data management and analytics package that engaged in a variety of applications all around the world, with countries mainly in Africa and Asia. This data aggregation and management tool equipped with advanced charts, geographic information system (GIS), pivots, and dashboards make it easy for the software to dynamic data visualization. It facilitates diverse applications (25, 26). Including (a) Health Information systems; (b) COVID-19 surveillance; (c) COVID-19 vaccine; (d) Education management systems; and (e) Tracking.

It records individual identities, and other socio-demographic details for accelerated case detection, case situation reporting, active surveillance, and the response to COVID-19. This model enables tracking techniques to trace out the suspected subsets.

## Dynamic dashboard models

The dashboard platforms are online dependant models which are exposed to substantial data processing in real-time. The integrated information from WHO, the Center for Disease Control and Prevention (CDC), the European Center for Disease Control and Prevention (ECDC), NXC, DXY, BNO are broadly used to track the COVID-19 statistics in real-time by International dashboards providers such as Johns Hopkins University dashboard, Microsoft Bing COVID-19, CDC, WHO The New York Times and worldometer.info (27, 28). GitHub avails the raw data to the general public (29) which ensures the access of COVID-19 statistics to the researchers. Data analyzing dashboards in LMICs like Sri Lanka which are facilitated by many domains such as HPB (Health promotion bureau) (30), *Arimac Digital* (31)*, Presidential secretariat* (32) are giving real-time case dynamics, death rate comparison, disease growth rate compared with other countries and district wise case distribution. These dashboards enable citizens to escape fake updates and causeless panic while receiving confidential updates from legal authorities.

### AI-enabled big data analysis and computer vision

Technology can provide an innovative solution even in the medical field from assisting in diagnosis, screening, and individual risk assessment. The AI-based big data analysis can either be used to assist risk assessment through case tracking and modeling or to improve the prognosis. Successful forecasting AI tools and algorithms (33) requires a bulk of data for training and validation to eliminate biases. Therefore, AI has not yet been impactful enough to solely provide robust solutions for forecasting outbreaks (34). Due to lack of raw data, noisy social media data, outliers, and big data hubris, most forecasting models used established epidemiological models, being called the SIR model (Susceptible, Infectious, and/or Recovered) (33) for disease forecasting. In addition, AI would potentially contribute to finding suitable vaccines (35–37). Few vaccines have been discovered in the recent past and quite a few of them are approved by WHO for emergency usage. WHO has approved to use of *Pfizer/BioNTech, AstraZeneca-SK Bio, Serum Institute of India, Janssen, Sinopharm COVID-*19, and *Moderna* vaccines for emergency use (38). However, clinical trials for these vaccines have finished and mass vaccinations and booster programs have started.

Healthcare workers (HCWs) and authorities are using AI-powered tools to adjunctively identify the target groups using AI computed tomography (CT) scanners and Temperature scanners [*iThermo*] after a standard test for diagnosis. RT-PCR (reverse transcriptase-polymerase chain reaction) fails to identify the asymptomatic cases by 39–61% (39, 40) and PCR tests consume days to identify the infection. Li et al. observed that the accuracy of CT imaging (98%) could be significantly higher than PCR (71%) (41). Hence, artificial intelligence-based CT scanning was established in Shanghai, China. A start-up called “*Infervision*” based in Beijing created software using artificial intelligence in CT Images. *Ping smart healthcare* unveiled a smart image reading system that reads CT images in 10s with more than 90% confidential rate whereas manual CT diagnostics can take up to 15 min (42). However, both RT-PCR and AI-enabled CT scan does not pose a serious impact on identifying infected patients. But rather it helps the health staff to diagnose and isolate patients from the crowd without major spread.

### Wearable data analytics and IoT

Wearable and implantable medical devices (IWMDs) use micro or nanosensors or implantable microchips for continuous health monitoring and treatments. These burgeoning wearable technologies hold significant potential to provide savings in terms of monetary cost and lives. However, externally wearable sensors are easy to use and can be worn flexibly. If any sensor detects abnormalities in physiological metrics, the device automatically alerts the patient through Bluetooth/Wi-fi to the mobile app and simultaneously communicates with the cloud through data fusion. It enables health professionals and caretakers to monitor remotely and take care of the patients in real-time. “Scripps research translational institute” in partnership with “*CareEvalution*” released the app-based solution, *DETECT*, which analyses wearable data shared by users to identify the spread of viral illness. These solutions are more desirable for in-home quarantine, to transfer the physiological parameters using electronic health records (EHR) without crowding test centers. “*Apple health Check-up Siri”*, “*Give me guidance”*, and “*Alexa daily check* “are a few examples that are providing the required functionality.

### Telehealth during the pandemic

Telehealth uses two-way media interactions to connect health professionals and patients from different geospatial locations who are often distant. Dr. *Ceasor Dhavaherian*, the chief medical officer of Telehealth company, *Carbon health*, says that telemedicine is an implication of healthcare services into virtual interactions assisted with devices whether using storing information in “*Apple watch*” or remote stethoscope like “*EKO Health*” or some of the other at-home pressure cuffs and connected scales. So, telehealth solutions are ahead of general health care systems, providing health care services from a safer distance. In a way, the best outcome of telehealth during the pandemic is that a doctor/physician can triage the suspects using physical symptoms without incurring patient in-hospital clustering. Hence, the telehealth solutions evolved during the pandemic crisis. However, several factors accompany the interventions in telehealth systems implementation.

### Robotics in healthcare

Autonomous robots are using cutting-edge telepresence technology to help to combat the spread of the COVID-19 virus. Robots have been used to deliver medicines, and foods, disinfect rooms, examine, treat suspected patients, and prevent staff from overexposing to the virus.

#### Delivery

The need for delivery robots is largely in demand for hospitals and food deliveries as the world has been confined to their homes without any means of supply. So, the demand for delivery robots is now on a spike. Beijing-based *Zhen robotics* is used to deliver foods, patrolling around the malls for those who are not wearing masks. Ezhou hospital has incorporated a robot chef in the kitchen for food preparation and serving. University of Michigan's start-up, *Refraction AI*, developed “*REV-1*” in food delivery as a pilot deployment for approximately 500 customers in corona use-cases. In another instance, the autonomous mobile robot “*Phollower 100*” by the company *Photoneo* was used for the safe distribution of medicines or auxiliary medicaments in the hospital quarantine zone.

#### Screening and treatment

The first COVID-19 patient who was treated with a robot equipped with a stethoscope and the virtual screen was in Seattle, USA. The robot was used for a man in isolation, used to take his vitals and communicate with the doctor on the console. China used hand sanitizer dispensing robots in their cities, refilled with disinfectants. A patrol robot in Shenyang, China, checks the body temperature and disinfects people and spaces. The operators on the mobile scooters control the robot. Tunisian police deployed a robot equipped with infrared, thermal imaging cameras, and an alarming system.

#### Awareness

A start-up company “*Asimov Robotics”* in India has launched two robots for the sake of spreading awareness, and distributing hand sanitizers and facemasks.

#### Interactive

“*Zorabots”* from Belgium designed a robot intended for elderly people to communicate with loved ones from the comfort of their home in a safe environment that keeps the most vulnerable groups from infection (43).

#### Disinfecting spaces

The health-threatening COVID-19 virus highlights the potential for finding solutions to disinfect transmission surfaces and bio-contaminated air. Hospital-acquired infection (HAIs) is a new threat in hospitals as the transmissions happen during hospital visits. The UV disinfection robotics systems are emerging to implement disinfection in hospitals, healthcare facilities, airports, and shopping malls. Robotics firms such as “*blue ocean robotics”*, “*UVD Robots”* and “*Kenex*” are at their peak to deploy UV-Light-based virus-fighting robots in hospital facilities all around the world. These robots are controlled using remote technology and refilled with sanitizers.

### Blockchain in healthcare

States and hospitals across many countries still have conflicting data on the number of ventilators, PPE, medications, and hospital/ICU beds in the supply chain, their locations, who is allowed to share them, and who is in the greatest need (44, 45). The information flow is fragmented and disorganized, making it extremely difficult for government agencies to respond to the needs during the pandemic efficiently. It is critical to have a single consolidated and accurate picture of real-time supply and demand information for these critical equipments in order to coordinate and effectively handle this issue. The combined capabilities of digital technologies like as Blockchain, Cloud, and AI may be leveraged to build a unified, trustworthy, and transparent view of supply and demand data across numerous stakeholders, locations, and legal organizations (46). Following that, local governments could collaborate to rapidly and effectively distribute supplies as demand varied from state to state. Blockchain is a type of decentralized ledger technology that allows for the safe storing and transport of data (47). Because each transaction is recorded across each node on the network, no record can be changed retrospectively without affecting any following blocks in the chain. Decentralizing data recording improves security and stability in data ownership and administration, as well as financial and logistical traceability in complicated healthcare supply chains (48).

## Role of social media in publicizing government safety measures

Education, public engagement, and technological literacy are the crucial elements that the general public considers while responding to the pandemic outbreak. Constituent public awareness was influenced through the press and media releases regardless of the truth. However, the resource-constrained subset of the population could still not access the real-time updates on time. But on the other hand, being overexposed to social media and fake online resources may result in psychological distress in some individuals, besides infection. Social media becomes one of the main sources of news online with 2.4 billion internet users, among them 64.5% receive breaking news from the sites like Instagram, Facebook, Twitter, WhatsApp, and Snapchat among others. It's estimated that 90.4% of millennials, 77.5% of generation Xers, and 48.2% of baby boomers are active social media users (49, 50). This international fact-checking network's (IFCN) WhatsApp bot uses machine learning tools to deliver the information (51). WhatsApp announced a $1 million grant for ICFN fact-checking ventures to report the rumors spreading *via* WhatsApp and SMS (52).

## COVID-19 digital responses

The sudden outbreak unleashed enormous digital solutions and potential mitigation strategies. Digital tools include the use of radio technology, television, mobile phones, blockchain, AI, unmanned aerial vehicles (UAV) drones, and geographic information systems (GIS) that enables internet and satellite technologies to provide timely solutions. Self-diagnosis, teleconsultation, information dissemination, case surveillance, reminders, laboratory data management, and track records are considerable features of digital response. Table 1 synthesizes digital health platforms that are effectively implemented in a few developing countries to help mitigate the spread. Tabulated solutions are some of the premier tools used by a few developing countries for the COVID-19 emergency response. These tools have enhanced the accessibility of healthcare to all citizens. The solutions can be re-established in other developing countries through careful assessment.

**Table 1:** List of digital solutions for countries to combat COVID-19.

**Usage**	**Function**	**Solution name**	**Company name**	**Reference**
Surveillance	Cloud-based solution for monitoring people during self-isolation with suspected or confirmed COVID-19 infection	COVID-19 Self-Isolation Monitoring and Notification	HN Consultants Ltd	(53)
	Cross border tracing tool enables to access personal records through the international borders	Journey solution	Inter-Governmental Agency for Development	(54)
	Hyperlocal heat map for the spatial dynamics of the at-risk population who are much more vulnerable to COVID-19 complications	Spatial dynamics of at-risk populations (Risk Profiles)	Fraym	(55)
	A field data collection tool during public health emergencies can be used for case detection, contact tracing, and visualization of transmission chains	Go. Data	Pan American Health Organization /World Health Organization	(56–58)
	‘Web-based & mobile app real-time traveler's data reporting at airport entry points. The data is then used for real-time evidence-based decision making	Traveler's Surveillance MIS (TSMIS)	USAID—GHSC PSM	(59)
	A platform that ensures active surveillance and tracing isolation. It groups the patients based on the geographical location of pathology and assigns health care staff to each patient	ADILIFE PLATFORM	ADITECH SRL	(60)
Prevention	A web-based learning platform to engage, train the healthcare workers, families about the prevention measures	Special Olympics	Special Olympics International	(61)
	Mobilizing tablet equipped female health workers in the villages of Punjab and Sind	LifesavHERs: how tech-enabled frontline health workers are combating COVID-19	doctHERs	(62)
	Maximize the usage of ICT enabled radio to disseminate knowledge, share health practices, and change the attitudes of rural sub-Saharan communities	Farm Radio	Farm radio international	(63)
	Crisis response platform that continually updates COVID-19 info on any mobiles and provides live, automated health coach, remote assessment & triage	Cell-Med COVID-19 Crisis Response	Cell-Ed	(64)
	An end user-focused app platform designed to share information, sending reminders for timely uptake of health services during COVID-19.	HERA App	HERA Inc.	(65)
Diagnosis	Highly configurable software application platform to capture diagnosis results, trigger and manage referrals, communicate with patients and health workers, and share data with other systems including DHIS2	Mango for diagnosis support	Greenmash	(66)
	Using the web, app, and messaging platforms, people can use diagnosis to assess risk, a guide to decision making, subscribe to alerts, find nearby testing sites and health services	COVIDcheck	The Commons Project	(67)
	Decision supporting and telemedicine app to train, assist frontline health workers, and for data analytics. Frontline Health workers may use it for risk assessment, decision support & provider-to-provider teleconsultation.	Intelehealth	Intelehealth	(68)
	An open-source mobile e-health system designed for case detection, contact tracing and follow up, laboratory data management, case management, and data analytics	Surveillance Outbreak Response Management and Analysis System (SORMAS)	Helmholtz Center for Infection Research (HZI)	(58)
	ODK is an open-source platform that has been used for surveillance, rapid diagnostics, and vaccine trials	ODK	Nafundi	(58, 69)
Treatment	WelTel is a secure, web-based, communication platform that uses bi-directional SMS for maximum reach while escalating to voice and video outreach when appropriate.	WelTel	WelTel Incorporation	(70)
	This module intended to provide preventive measures, case management, risk management, mental, psychological support, and mass vaccination	COMPASS	Save the children (UK)	(71)
	Digital platform for rural/remote settings in LMICs: contact tracing; symptom checker/chatbot; HCW remote training and implementation; COVID-19 risk-scoring; last-mile logistics for delivery of medicines; micro-insurance	reah52 COVID-19 response	reach52	(72)
	A two-way, mobile phone-based real-time communication system that uses basic text messaging, or SMS, to connect national health authorities and front-line health workers.it operates on simple talk-and-text mobile devices—no smartphone or tablet required. Equip health workers with information, supplies, and support	mHero	IntraHealth International	(73)
	A directory of public and private testing sites and isolation centers, hotline numbers in Africa (LMIC). It prepares all citizens to know the whereabouts of treatment/Isolation centers	NaviHealth	mDoc Healthcare	(74)

## Challenges and opportunities

Digitalization brings challenges in disguise, despite being hailed as a source of development. Digitalization produces timely solutions and is important during the pandemic. However, individuals may be reluctant to the change and have several concerns. In particular, the employees in an organization may expose to severe pushbacks once the pandemic is over. Reasonably, they may have a fear of losing jobs, changes in their roles, being unable to adapt to the new environment, and taking additional responsibilities if digitalisation demands fewer human interactions. Therefore, organizations and entities should be prepared for these perceived consequences and should plan solutions to futureproof their working solutions and staffing needs.

## Conclusion

The COVID-19 pandemic imposed seamless challenges on the healthcare sector globally. Digital technologies can provide an accurate and robust solution for the ongoing and upcoming outbreaks, with measures to support the healthcare sector. The technology brings in new perceptions and hope for the betterment of the community with utmost effectiveness. These digital healthcare solutions provide accelerated case detection, constant surveillance, access, advanced decision-making, and virtual consultations while improving the quality of services. The concerned authorities and decision-makers should understand the basic functionality, intrinsic capabilities, opportunities, and possibilities of these solutions in all dimensions. A rigorous and honest assessment of the suitability and reliability of how these solutions integrate with the existing infrastructure of the country is also necessary. Furthermore, digital health solutions require extensive collaboration and support from disparate entities and the public.

## Author contributions

NN, BH, BS, BR, and VP contributed to the conception and design of the study. NS, SV, KS, JS, and SI organized the database and wrote the first draft of the manuscript. NS, SV, KS, SI, AS, HK, KN, DS, and VP wrote sections of the manuscript. PC, BH, BR, and BS critically reviewed and edited the manuscript. All authors contributed to manuscript revision, read, and approved the submitted version.

## Conflict of interest

The authors declare that the research was conducted in the absence of any commercial or financial relationships that could be construed as a potential conflict of interest.

## Publisher's note

All claims expressed in this article are solely those of the authors and do not necessarily represent those of their affiliated organizations, or those of the publisher, the editors and the reviewers. Any product that may be evaluated in this article, or claim that may be made by its manufacturer, is not guaranteed or endorsed by the publisher.

## References

[1] Yang X Yu Y Xu J Shu H Xia J Liu H. Clinical course and outcomes of critically ill patients with SARS-CoV-2 pneumonia in Wuhan, China: a single-centered, retrospective, Observational Study. Lancet Respir Med. (2020) 8:475–81. 10.1016/S2213-2600(20)30079-5 32105632 PMC7102538

[2] COVID Live Update: 171 468 758 Cases and 3 565 021 Deaths from the Coronavirus - Worldometer. Worldometer.info. Available online at: https://www.worldometers.info/coronavirus/?fbclid=IwAR35ZFiRZJ8tyBCwazX2N-k7yJjZOLDQiZSA_MsJAfdK74s8f2a_Dgx4iVk (accessed June 1, 2021).

[3] Ma X Vervoort D. Critical care capacity during the COVID-19 pandemic: Global availability of intensive care beds. J Crit Care. (2020) 58:96–7. 10.1016/j.jcrc.2020.04.012 32408107 PMC7194590

[4] Mannucci E Silverii GA Monami M. Saturation of critical care capacity and mortality in patients with the novel coronavirus (COVID-19) in Italy. Trends Anaesth Crit Care. (2020) 33:33–4. 10.1016/j.tacc.2020.05.002 PMC7244438 38620165

[5] Murthy S Leligdowicz A Adhikari NKJ. Intensive care unit capacity in low-income countries: a systematic review. PLoS ONE. (2015) 10:1–12. 10.1371/journal.pone.0116949 25617837 PMC4305307

[6] Hamadani JD Hasan MI Baldi AJ Hossain SJ Shiraji S Bhuiyan MSA. Immediate impact of stay-at-home orders to control COVID-19 transmission on socioeconomic conditions, food insecurity, mental health, and intimate partner violence in Bangladeshi women and their families: an interrupted time series. Lancet Glob Heal. (2020) 8:e1380–9. 10.1016/S2214-109X(20)30366-1 32857955 PMC7447230

[7] Mahmud M Riley E. Household response to an extreme shock: Evidence on the immediate impact of the Covid-19 lockdown on economic outcomes and well-being in rural Uganda. World Dev. (2021) 140:105318. 10.1016/j.worlddev.2020.105318 34548741 PMC8446716

[8] Marazziti D Stahl SM. The relevance of COVID−19 pandemic to psychiatry. World Psychiatry. (2020) 19:261–261. 10.1002/wps.20764 32394565 PMC7215065

[9] Rossi R Socci V Talevi D Mensi S Niolu C Pacitti F. COVID-19 pandemic and lockdown measures impact on mental health among the general population in Italy. Front Psychiatry. (2020) 11:7–12. 10.3389/fpsyt.2020.00790 32848952 PMC7426501

[10] Asahi K Undurraga EA Valdés R Wagner R. The effect of COVID-19 on the economy: Evidence from an early adopter of localized lockdowns. J Glob Health. (2021) 11:05002. 10.7189/jogh.10.05002 33643635 PMC7897430

[11] Nicola M Alsafi Z Sohrabi C Kerwan A Al-Jabir A Iosifidis C. The socio-economic implications of the coronavirus pandemic (COVID-19): a review. Int J Surg. (2020) 78:185–93. 10.1016/j.ijsu.2020.04.018 32305533 PMC7162753

[12] Digital health and COVID-19. Bull World Health Organ. (2020) 98:731–2. 10.2471/BLT.20.021120 33177768 PMC7607467

[13] Gunasekeran DV Tseng RMWW Tham YC Wong TY. Applications of digital health for public health responses to COVID-19: a systematic scoping review of artificial intelligence, telehealth and related technologies. npj Digit Med. (2021) 4:36–41. 10.1038/s41746-021-00412-9 33637833 PMC7910557

[14] Kapoor A Guha S Kanti Das M Goswami KC Yadav R. Digital healthcare: the only solution for better healthcare during COVID-19 pandemic? Indian Heart J. (2020) 72:61–4. 10.1016/j.ihj.2020.04.001 32534691 PMC7151273

[15] World Health Organization. Operational Planning Guidelines To Support Country Preparedness and Response. Geneva: World Health Organization (2020).

[16] Elementor #4517. Collage of Community Physicians of Sri Lanka. Available online at: https://ccpsl.org/elementor-4517/ (accessed May 31, 2021).

[17] Sri Lanka's Highly Efficient Public Health Sector Faces New Private Competition Sri Lanka 2016. Oxford Business group. Available online at: https://oxfordbusinessgroup.com/overview/vital-signs-highly-efficient-public-health-sector-faces-new-private-competition (accessed June 1, 2021).

[18] Bar-On YM Flamholz A Phillips R Milo R. SARS-CoV-2 (Covid-19) by the numbers. Elife. (2020) 1:9. 10.7554/eLife.57309 32228860 PMC7224694

[19] Ham S. Prevention of exposure to and spread of COVID-19 using air purifiers: challenges and concerns. Epidemiol Health. (2020) 42:e2020027. 10.4178/epih.e2020027 32311865 PMC7340613

[20] Sayampanathan AA Heng CS Pin PH Pang J Leong TY Lee VJ. Infectivity of asymptomatic versus symptomatic COVID-19. Lancet. (2021) 397:93–4. 10.1016/S0140-6736(20)32651-9 33347812 PMC7836843

[21] Oran DP Topol EJ. Prevalence of asymptomatic SARS-CoV-2 infection : a narrative review. Ann Intern Med. (2020) 173:362–7. 10.7326/M20-3012 32491919 PMC7281624

[22] Vaudenay S. Centralized or decentralized? The contact tracing dilemma. Cryptol ePrint Arch. (2020). Available online at: https://eprint.iacr.org/2020/531

[23] House of Commons House of Lords Joint Committee on Human Counter-Extremism : Government Response to the Committee's Second Report of. (2020). Available online at: https://committees.parliament.uk/publications/2649/documents/26914/default/ (accessed May 15, 2021).

[24] Apple and Google partner on COVID-19 contact tracing technology - Apple. Apple.com. (2020). Available online at: https://www.apple.com/newsroom/2020/04/apple-and-google-partner-on-covid-19-contact-tracing-technology/ (accessed June 1, 2021).

[25] In Action. DHIS2. Available online at: https://dhis2.org/in-action/ (accessed May 15, 2021).

[26] DHIS2 for COVID-19 Surveillance: Sri Lankan use case - Resources - Ressources/User stories - DHIS2 Community. DHIS2. (2020). Available online at: https://community.dhis2.org/t/dhis2-for-covid-19-surveillance-sri-lankan-use-case/38516 (accessed May 15, 2021).

[27] COVID-19 deaths and cases: how do sources compare? - Our World in Data. ourworldindata.org. (2020). Available online at: https://ourworldindata.org/covid-sources-comparison (accessed May 31, 2021).

[28] Microsoft Launches Easy-to-Use Coronavirus Tracker Map. inputmag.com. Available online at: https://www.inputmag.com/tech/microsoft-launches-easy-to-use-coronavirus-tracker-map (accessed May 31, 2021).

[29] GitHub - CSSEGISandData/COVID-19: Novel Coronavirus (COVID-19) Cases provided by JHU CSSE. Github. Available online at: https://github.com/CSSEGISandData/COVID-19 (accessed May 15, 2021).

[30] HPB. Live Updates on New Coronavirus (COVID-19) Outbreak. HPB. Available online at: https://www.hpb.health.gov.lk/en (accessed May 15, 2021).

[31] Coronavirus (COVID-19) Sri Lanka - Analytics Dashboard. arimaclanka.com. Available online at: https://covid19-dashboard.arimac.digital/ (accessed June 2, 2021).

[32] COVID-19 Dashboard – Presidential Secretariat of Sri Lanka. presidentsoffice.gov.lk. Available online at: https://www.presidentsoffice.gov.lk/index.php/covid-19-dashboard/ (accessed June 2, 2021).

[33] Wang L Zhou Y He J Zhu B Wang F Tang L. An Epidemiological Forecast Model Software Assessing Interventions on COVID-19 Epidemic in China. medRxiv. (2020). Available online at: https://www.medrxiv.org/content/10.1101/2020.02.29.20029421v1.full.pdf (accessed May 15, 2021).

[34] Naudé W. Artificial intelligence against COVID-19: an early review. IZA Discuss Pap. (2020) 13110. 10.2139/ssrn.3568314

[35] Keshavarzi Arshadi A Webb J Salem M Cruz E Calad-Thomson S Ghadirian N. Artificial intelligence for COVID-19 drug discovery and vaccine development. Front Artif Intell. (2020) 3:1–13. 10.3389/frai.2020.00065 33733182 PMC7861281

[36] Chandra Kaushik A Raj U. AI-driven drug discovery: a boon against COVID-19? AI Open. (2020) 1:1–4. 10.1016/j.aiopen.2020.07.001

[37] Zhou Y Wang F Tang J Nussinov R Cheng F. Artificial intelligence in COVID-19 drug repurposing. Lancet Digit Heal. (2020) 2:e667–76. 10.1016/S2589-7500(20)30192-8 32984792 PMC7500917

[38] WHO Lists Additional COVID-19 Vaccine for Emergency Use and Issues Interim Policy Recommendations. World Health Organisation. (2021). Available online at: https://www.who.int/news/item/07-05-2021-who-lists-additional-covid-19-vaccine-for-emergency-use-and-issues-interim-policy-recommendations (accessed June 1, 2021).

[39] CDC. Real-Time RT-PCR diagnostic panel. Centers Dis Control Prev. (2020). CDC-006-00:1–80.

[40] Kucirka LM Lauer SA Laeyendecker O Boon D Lessler J. Variation in false-negative rate of reverse transcriptase polymerase chain reaction-based SARS-CoV-2 tests by time since exposure. Ann Intern Med. (2020) 173:262–7. 10.7326/M20-1495 32422057 PMC7240870

[41] Li X Zeng W Li X Chen H Shi L Li X. CT imaging changes of corona virus disease 2019(COVID-19): a multi-center study in Southwest China. J Transl Med. (2020) 18:4–11. 10.1186/s12967-020-02324-w 32252784 PMC7132551

[42] China Uses AI in Medical Imaging to Speed Up COVID-19 Diagnosis. BioWorld. (2020). Available online at: https://www.bioworld.com/articles/433530-china-uses-ai-in-medical-imaging-to-speed-up-covid-19-diagnosis (accessed May 16, 2021).

[43] Robots Keeping Elderly Belgians Connected With Loved Ones During Coronavirus. E&T Magazine. Engineering and technology. (2020). Available online at: https://eandt.theiet.org/content/articles/2020/03/robots-keep-elderly-connected-with-loved-ones-during-coronavirus/ (accessed May 16, 2021).

[44] Cooper N. Editorial: blockchain in health care. Front Blockchain. (2022) 4:830459. 10.3389/fbloc.2021.830459

[45] Sahal R Alsamhi SH Brown KN O'Shea D Alouffi B. Blockchain-based Digital Twins collaboration for smart pandemic alerting: decentralized covid-19 pandemic alerting use case. Comput Intell Neurosci. (2022) 2022:1–14. 10.1155/2022/7786441 35035466 PMC8759442

[46] Alsamhi SH Lee B. Blockchain-empowered multi-robot collaboration to fight COVID-19 and future pandemics. IEEE Access. (2021) 9:44173–97. 10.1109/ACCESS.2020.3032450 34786312 PMC8545252

[47] Ng WY Tan T-E Movva PV Fang AH Yeo K-K. Blockchain applications in health care for covid-19 and beyond: A systematic review. Lancet Digital Health. (2021) 3:e819–29. 10.1016/S2589-7500(21)00210-7 34654686 PMC8510632

[48] Alsamhi SH Lee B Guizani M Kumar N Qiao Y Liu X. Blockchain for decentralized multi-drone to combat Covid−19 and future pandemics: Framework and proposed solutions. Transact Emerg Telecommun Technol. (2021) 32. 10.1002/ett.4255

[49] Hameed I Irfan BZ. Social media self-control failure leading to antisocial aggressive behavior. Hum Behav Emerg Technol. (2021) 3:296–303. 10.1002/hbe2.226

[50] Hussaini N Anuradha B Sachin K. Behavioural dynamics of teenagers exposed to social media : a case based enquiry of Indian youth. Psychol Educ. (2020) 57:499–504. 10.1007/s13312-020-1840-8

[51] Chat on WhatsApp with World Health Organization. Whatsapp. Available online at: https://api.whatsapp.com/send/?phone=41798931892&text=hi&app_absent=0 (accessed May 16, 2021).

[52] Coronavirus - WHO Health Alert Launch. Whatsapp.com. Available online at: https://www.whatsapp.com/coronavirus/who/?lang=en (accessed June 1, 2021).

[53] HN Consultants. COVID-19. (2020). Available online at: http://hncl.com/home/covid19 (accessed May 16, 2021).

[54] Journey Cross Border Immunization Tracking Tool. www.jembi.org. (2019). Available online at: https://www.jembi.org/Project/Journey-Cross-Border-Immunization-Tracking-Tool (accessed May 16, 2021).

[55] Machine Learning to Identify High-Risk COVID-19 Populations - Fraym. fraym. (2020). Available online at: https://fraym.io/machine-learning-to-identify-high-risk-covid-19-populations/ (accessed May 16, 2021).

[56] WHO. Go Data. Available online at: https://www.who.int/tools/godata (accessed May 16, 2021).

[57] PAHO/WHO. A Tool to Investigate Outbreaks Go.Data is Rolled Out for COVID-19 in Latin America. PAHO. Available online at: https://www3.paho.org/hq/index.php?option=com_content&view=article&id=15747:inician-en-mexico-la-puesta-en-marcha-de-go-data-en-america-latina-una-herramienta-para-investigar-covid-19-y-otros-brotes-epidemicos&Itemid=1926&lang=en (accessed May 16, 2021).

[58] CDC. Guide to Global Digital Tools for COVID-19 Response. Atlanta, GA: CDC (2020).

[59] CHE LOGIN. http://id.lmis.gov.pk/. Available online at: http://id.lmis.gov.pk/che/application/che/index.php (accessed May 16, 2021).

[60] Covid-19 - ADiLife. www.adilife.net. Available online at: https://www.adilife.net/en/covid-19/ (accessed May 16, 2021).

[61] Special Olympics Online Learning Portal. learn.specialolympics.org. Available online at: https://learn.specialolympics.org/ (accessed May 16, 2021).

[62] (20+) doctHERs. Facebook. https://www.facebook.com/doctherspk/. Available online at: https://www.facebook.com/doctherspk/ (accessed May 16, 2021).

[63] Farm Radio International. https://farmradio.org/. Available online at: https://farmradio.org/ (accessed May 16, 2021).

[64] Solutions - Cell-Ed. www.cell-ed.com. Available online at: https://www.cell-ed.com/solutions/ (accessed May 16, 2021).

[65] Hera Project – Medak. https://project-hera.com/. Available online at: https://project-hera.com/ (accessed May 16, 2021).

[66] mHealth. Surveillance Monitoring & Reporting Solutions. Greenmash. http://greenmash.com/. Available online at: http://greenmash.com/ (accessed May 16, 2021).

[67] COVIDcheck. https://www.covidcheck.org/en/. Available online at: https://www.covidcheck.org/en/ (accessed May 16, 2021).

[68] Intelehealth. https://www.intelehealth.org/. Available online at: https://www.intelehealth.org/ (accessed May 16, 2021).

[69] Open Data Kit. https://opendatakit.org/. Available online at: https://opendatakit.org/ (accessed May 16, 2021).

[70] Virtual Care (mHealth) for COVID-19. Weltelhealth.com. Available online at: https://covid.weltelhealth.com/covid19 (accessed June 1, 2021).

[71] COMPASS Modules. Save the Children. Available online at: https://compass.savethechildren.org.uk/ (accessed June 1, 2021).

[72] Covid-19 Solutions. reach52. Available online at: https://reach52.com/covid-19-solutions/ (accessed June 27, 2022).

[73] COVID-19 Response. Intrahealth mHero. mHero.org. Available online at: https://www.mhero.org/covid-19-response (accessed June 1, 2021).

[74] NaviHealth.ai. Health Directory Find the Best Doctors and Hospitals in Africa – Find a Hospital – Doctor Reviews – Doctor Near You. navihealth.ai. Available online at: https://www.navihealth.ai/covid-19 (accessed June 1, 2021).

